# Ischemia-Modified Albumin: Origins and Clinical Implications

**DOI:** 10.1155/2021/9945424

**Published:** 2021-07-19

**Authors:** Alla Shevtsova, Iuliia Gordiienko, Viktoriia Tkachenko, Galyna Ushakova

**Affiliations:** ^1^Oles Honchar Dnipro National University, 72 Gagarin Ave, Dnipro 49010, Ukraine; ^2^Dnipro State Medical University, 9 Vernadsky Str., Dnipro 49044, Ukraine

## Abstract

Albumin is one of the most abundant proteins in the body of mammals: about 40% of its pool is located in the intravascular space and the remainder is found in the interstitial space. The content of this multifunctional protein in blood is about 60-65% of total plasma proteins. A decrease in its synthesis or changes of functional activity can destabilize oncotic blood pressure, cause a violation of transporting hormones, fatty acids, metals, and drugs. Albumin properties change under ischemic attacks associated with oxidative stress, production of reactive oxygen species, and acidosis. Under these conditions, ischemia-modified albumin (IMA) is generated that has a reduced metal-binding capacity, especially for transition metals, such as copper, nickel, and cobalt. The method of determining the cobalt-binding capability of HSA was initially proposed to evaluate IMA level and then licensed as an ACB test for routine clinical analysis for myocardial ischemia. Subsequent studies have shown the viability of the ACB test in diagnosing other diseases associated with the development of oxidative stress. This review examines recent data on IMA generation mechanisms, describes principles, advantages, and limitations of methods for evaluation of IMA levels, and provides detailed analysis of its use in diagnostic and monitoring therapeutic efficacy in different diseases.

## 1. Introduction

Albumin is one of the most abundant proteins in the human body, with about 40% circulating in the bloodstream. It is also a significant component of most extracellular fluids, including lymph, interstitial, and cerebrospinal fluids [1]. Constant redistribution of the protein is achieved through the dynamic exchange of its intravascular and extravascular pools [2]. Albumin is synthesized in the liver at a rate of 10-12 g/day, accounting for about 25% of total proteins [3]. In addition, smaller amounts of albumin can be produced in extrahepatic tissues, such as kidneys, pancreas, intestines, lymph, mammary glands, reproductive tissues, and brain [4–6]. This multifunctional globular protein has a molecular weight of approximately 66-69,000 Dа, with minor variations among species. There is 83% to 88% amino acid homology among albumin molecules of many veterinary species [7–9].

**Figure 1:**
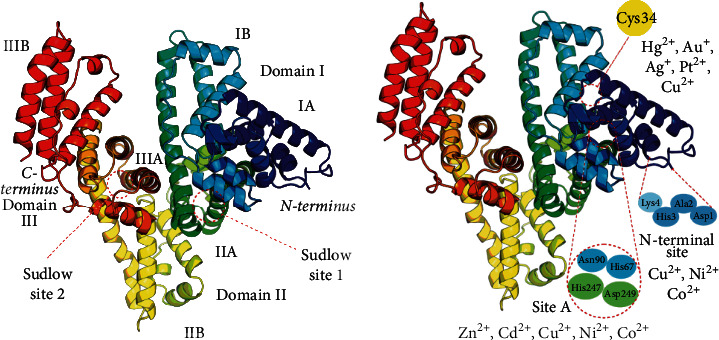
Structure of human serum albumin. (a) The molecule consists of a single polypeptide chain; about half of its length is an *α*-helix. The albumin structure comprises three homologous domains: I, marked in blue and cyan; II, green and yellow; III, orange and red. Each domain contains two subdomains, A and B, and two sites to bind hydrophobic molecules (Sudlow sites 1 and 2). (b) Sites for binding transition metal ions: N-terminal site, Cys34, and site А (multimetal binding site). Site B is not shown because its exact position is unknown.

The single polypeptide chain of human serum albumin (HSA) comprises 585 amino acid residues that form nine loops connected by 17 intramolecular disulfide bonds. HSA contains three homologous domains (I, II, and III), each split into A and B subdomains. The multifunctionality of albumin results from the presence of multiple binding sites. HSA contains Sudlow sites 1 and 2 that play a significant role in transporting hydrophobic molecules (Figure 1(a)), seven fatty acid-binding sites (FA1-7), heme-binding site, numerous small ligand-binding sites, and four metal-binding sites, including sites A and B, N-terminal site (NTS), and Cys34 (Figure 1(b)) [10–12].

Metal-binding sites differ in affinity for their ligands. Site A can bind several metals, including Zn^2+^, Cd^2+^, Ni^2+^, Co^2+^, and Cu^2+^; it is often referred to as a multimetal binding site A [13]. Site B has a high affinity for cadmium and so has been generically labeled as a secondary multimetal binding site or cadmium site B [10]. NTS has the strongest affinity for cobalt, copper, and nickel ions, and Cys34—for gold and platinum [11]. The cysteine residue at position 34 has a free SH group that does not form a disulfide bond and is believed to be a target site for redox modifications of albumin [14].

Posttranslational changes, including oxidation, glycation, carbamylation, nitrosylation, guanylation, dimerization, and truncation, form numerous albumin variants that appear or become more abundant in the blood as a result of metabolic changes associated with various diseases [15–17]. Of particular significance is ischemia-modified albumin, a subject of clinical and laboratory studies discussed in this review.

## 2. Generation of Ischemia-Modified Albumin

**Figure 2:**
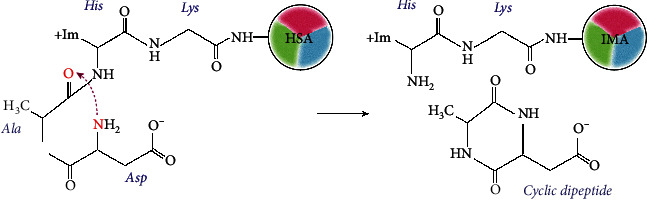
ІМА formation through dipeptide cleavage. A nucleophilic attack by the *α*-amino nitrogen on the carbonyl of Ala2-His3 peptide bond cleaves and releases the cyclic dipeptide. The truncated NTS cannot bind transition metal ions [20].

Albumin properties incur some changes under ischemic attacks associated with oxidative stress, production of reactive oxygen species (ROS), and development of acidosis [15, 18, 19]. The N-terminal sequence of HSA (Asp1-Ala2-His3-Lys4) is very susceptible to biochemical modifications and degradation induced by oxidative stress. Consequently, the affinity of NTS to transition metals, especially to cobalt, is reduced. This variant of albumin was called ischemia-modified albumin (IMA) [11, 13, 16]. Some models were proposed to explain the IMA formation. One of them is an autodegradation of NTS, the scheme of which is shown in Figure 2.

According to this model, the *α*-amino group of Asp1 exhibits nucleophilic properties caused by the dissociation of a carboxyl group and the release of a proton. A nucleophilic attack of Asp1 amine nitrogen on the carbonyl of the peptide bond between Ala2 and His3 leads to its cleave and release of a cyclic dipeptide. As result, truncated albumin cannot bind transition metal ions [20].

**Figure 3:**
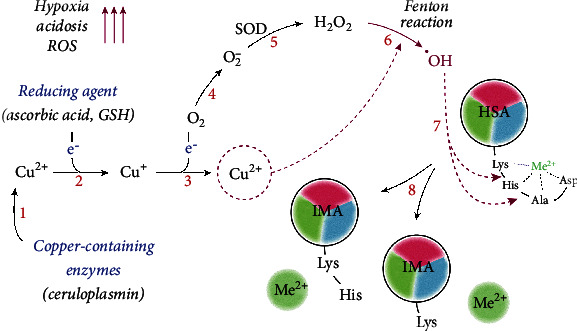
The mechanism of ІМА formation driven by oxidative stress. Tissue hypoxia and activation of anaerobic glycolysis induce acidosis and release Cu^2+^ ions from copper-containing proteins, such as ceruloplasmin (1). In the presence of reducing agents, e.g., ascorbic acid, Cu^2+^ is reduced to Cu^+^ (2), followed by the formation of superoxide anion O^−^_2_ (3-4). Superoxide dismutase (SOD) catalyzes the dismutation of superoxide O^−^_2_ to hydrogen peroxide H_2_O_2_ (5), which, in the presence of Cu^2+^, undergoes the Fenton reaction with the formation of hydroxyl radicals ^·^OH (6). These radicals contribute to the degradation of NTS (7) and IMA formation (8), which cannot bind Cu^2+^ and other metal ions.

Another model of ІМА formation is based on the generation of reactive oxygen species during the Fenton reaction. According to this model, ischemia results in the acidosis and release of Cu^2+^ from weak binding sites on circulating proteins and peptides. In the presence of reducing agents (for example, ascorbic acid), free Cu^2+^ is converted to Cu^+^, which can then react with O_2_ and generate superoxide radicals. During this reaction, Cu^+^ is oxidized to Cu^2+^, and albumin N-terminus scavenges these ions. The superoxide radicals are converted to hydrogen peroxide (H_2_O_2_) by superoxide dismutase, and H_2_O_2_ is then degraded by catalase or converted to hydroxyl free radicals in the Fenton reaction. These radicals can damage HSA, causing the removal of two or three N-terminal amino acids and releasing Cu^2+^. The steps of the above-mentioned process are repeated in a chain reaction [11, 21], and IMA rises rapidly following an ischemic attack. The stages of IMA formation by this mechanism are presented in Figure 3.

This postulated mechanism, although theoretically attractive, has not been borne out in practice. The half-life of HSA *in vivo* is about 3 weeks. HSA with a truncated NH_2_-terminus would presumably have similar *in vivo* half-life properties. However, clinical and experimental observations show that IMA returns to baseline rapidly after an ischemic cardiac event [22]. So, the modification of albumin to create IMA is transient and reversible, rather than a finite chemical alteration. Rapid regeneration of truncated albumin is unlikely. Bhagavan et al. showed that N-terminal amino acid sequencing for purified albumin had nontruncated NTS in six of seven ischemic individuals with high IMA and one nonischemic individual, and only one individual with high IMA had two missing amino acid residues from the N-terminal region [23]. Using synthetic peptides with altered first 2-12 amino acids of the HSA sequence, Bar-Or et al. revealed that IMA could be formed not only by truncation but by the acetylation of NTS [24]. However, this modification has not been demonstrated *in vivo*.

A different suggestion was made after the spectroscopic and thermodynamic determinations of three distinct binding sites for Co^2+^-ions in human serum albumin, where it was shown that A- and B-sites had greater avidity than the N-terminal binding site [25]. Fatty acids bind to albumin at one of the additional cobalt-binding sites with a negative allosteric interaction. Hypothetically, the release of fatty acids in myocardial ischemia results in their binding to albumin, reducing albumin's ability to take up cobalt [26]. Taking into account all of the above, we can note the following: currently, there is no generally accepted mechanism for the IMA formation; a common sign to all described mechanisms is a decrease in the affinity of albumin to transition metal ions.

IMA formation kinetics have been studied in patients with chronic stable angina undergoing percutaneous coronary intervention (PCI) considered a clinical model of myocardial ischemia-reperfusion. According to the results of these studies, blood IMA increased within 6-10 minutes following PCI; it remained high for about 6-12 hours and returned to normal after 12-24 hours in patients with positive exercise stress test and coronary artery disease [22, 27]. In short-term physical activity in athletes, the return of IMA levels to normal may take about 12 hours [28]. These data show that IMA quickly returns to the baseline level after ischemia. Because albumin has a half-life of about 19-20 days, it is plausible that albumin modification is only temporary or that IMA is rapidly eliminated from the body. IMA content also depends on the duration of ischemic events: its levels after prolonged ischemia (25-60 min) are much higher than levels observed after short-term (15-21 min) ischemia [11]. Thus, IMA kinetic features should be taken into account when conducting research and evaluating results.

## 3. Methods for Measuring ІМА

**Figure 4:**
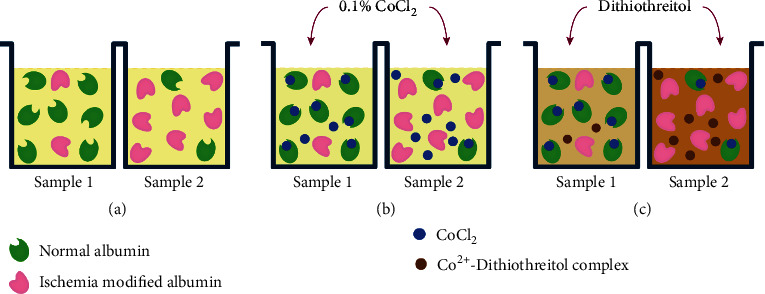
The scheme of the ACB test. Serum samples (100 *μ*L) are added in the wells of the microplate (a), then add 25 *μ*L of CoCl_2_ (b), incubate for 10 min, and then add dithiothreitol (c), which binds to free cobalt, staining the medium brown. The color intensity is proportional to the amount of free cobalt and the amount of IMA.

Bar-Or et al. were the first to develop an assay for ІМА determination based on measuring the degree of its interaction with metal ions, particularly, Co^2+^ [29]. Ischemia Technologies Incorporated (Denver, Colorado) used these findings to develop the Albumin Cobalt Binding test (ACB) that was approved in 2003 by the United States Food and Drug Administration (FDA) as a novel method for ruling out myocardial infarction. In principle, a known number of cobalt ions is added to a serum sample and binds to normal albumin but not to IMA. The remaining free cobalt ions react with dithiothreitol, upon its addition as a colorizing reagent, to form colored complexes that can be quantified spectrophotometrically. The IMA concentration is directly proportional to the concentration of the colored complex and, thus, the color intensity [23, 29]. A scheme of the ACB test is shown in Figure 4.

Many studies have shown that the ACB test has some limitations. First, conformational changes in albumin upon the fluctuation of pH, the presence of denaturing agents, chemicals, or medications can lead to inaccurate results of the assay [30–32]. Second, samples and reagents' lability need to be kept in mind. IMA's lability requires that the sample is measured within 2.5 hours of its collection or refrigerated/frozen until analysis. The dithiothreitol reagent and, hence, the entire kit are only stable for 14 days [33]. In addition, albumin has sites for binding fatty acids and other hydrophobic molecules that can mask cobalt-binding sites and, thus, cause an error in ACB test results [26, 34]. According to recent findings, fatty acids bind to the high-affinity FA2 site of albumin, leading to conformational changes that, via an allosteric mechanism, hinder albumin's ability to bind metal ions at site A and, partially, at site B [35]. Co^2+^ preferably binds to site B, then site A, and finally NTS [26, 36]. Hence, when using the ACB test, IMA values may correspond to albumin with increased levels of bound fatty acids; therefore, all result inferences should be made with these possibilities in mind [37]. Some researchers suggest that inaccuracies in ACB assays occur due to an increase in endogenous lactate in ischemic tissues. Negative correlations [38, 39], strong positive correlations [40], or lack of correlation [22, 41] have been established between these markers of ischemia. Therefore, IMA test results in patients with uncontrolled diabetes, chronic renal failure, sepsis, and other conditions associated with increased lactate levels and lactate acidosis should be evaluated cautiously.

One way to avoid the impact of acidic metabolic products on the level of measured IMA is using the buffer systems during ACB analysis [34, 42]. Total plasma albumin concentration should also be considered, especially in individuals with hypo- or hyperalbuminemia, to avoid misinterpretation of IMA values. The reason for such interference is related to the principle of the assay. When there are low albumin levels in a specimen, less cobalt binds to the protein, allowing a major quantity to react with DTT, and vice versa [43]. In this case, measurement errors can be avoided by calculating the IMA/albumin ratio (IMAR coefficient). This ratio is especially important, if the level of IMA is examined in other biological fluids, such as in urine or saliva. It is interesting to note that urine IMA in patients with diabetic nephropathy does not correlate with the level of albuminuria [42]. To correct IMA values for levels of serum albumin, Lippi et al. proposed the calculating of albumin-adjusted ischemia-modified albumin (AAIMA) levels using the following formula: (individual serum albumin concentration/median albumin concentration of the population) × IMA value [43]. The perspectives of determining AAIMA for the standardization of ACB results in clinical investigations have been demonstrated in some investigations [44, 45]. At last, a controlled temperature regime is required for the stability and reproducibility of the ACB test, as a 10°C temperature increase during incubation leads to a nearly 1.5-time increase in the measured value of IMA [46].

Several modifications have been made to this test, all of which differ in the amount and content of reagents. One such modification, named Cobalt Albumin Binding assay (CAB), was proposed by Lee et al. [34]. The authors proposed a buffer system for the CAB assay, reduced the necessary sample volume, and optimized assay time, ratio of the reagents, and some other factors that may affect the assay outcomes. One of their findings indicated that the CAB allows to detect overall structural changes in albumin; therefore, it may be applicable in albumin-related research, including the quality control of albumin injections with reliable efficiency. Despite the described advantages of the CAB analysis technique, such as high sensitivity, resistance to changes in pH, and the influence of exogenous factors, some of its provisions are unclear, for example, how an increase in free FA in the blood of patients with myocardial ischemia affects the CAB assay results [47].

Some studies have used Cu^2+^ and Ni^+2^ instead of cobalt ions to assess human ІМА levels. A colorimetric method based on nickel-albumin binding properties (NAB) was described by da Silva et al. to measure ischemia-induced alterations in the binding capacity of HSA to exogenous nickel [48]. This assay involved such steps as ACB; only CoCl_2_ is replaced by an equivalent amount of nickel sulfate. The authors of this method found a significant correlation between ACB and NAB revealing their low specificity, with the sensitivity of NAB higher than ABC's [49]. Although this method correlated well with the ACB assay, its clinical applications must still be verified.

Based on the experimental model for evaluating the affinity of different metal ions to N-terminal peptide of HSA, Eom et al. developed an albumin copper-binding test (ACuB). They proposed to detect the Cu-HSA complex by the copper-specific fluorescent reagent Lucifer Yellow and selected the optimal conditions for analysis. According to their results, the ACuB is more specific and has a higher sensitivity than ACB, and it can be used to determine IMA in different animals, including bovine, rat, and human [50]. It should be noted that sites A and B are the potent secondary binding sites for transition metal ions, and free fatty acids can affect the Cu^2+^ and Ni^2+^ binding capacity of albumin in these sites [35]. Therefore, such tests can be used only in an excess of copper or nickel ions during the analysis. It was found molar ratio metal : albumin needs to be more than 2-3 to have reproducible results [51].

A common problem for all colorimetric methods is the lack of their standardization. Most authors have reported findings in absorbance units (ABSU, see Table 1), which often depend on experience of the investigator and sensitivity of the laboratory equipment [47, 51–53]. Some researchers have used IMA internal standards obtained in their laboratories [54]. These limitations explain partly the significant scatter in the results presented in the existing literature. Another reason for the variability is the lack of a unified IMA standard suitable for various methods, since there is still no consensus regarding the mechanism of IMA formation and its structural features.

Another group of methods for assessing IMA concentration in biological fluids is based on immunological reactions using antibodies to modified albumin. These methods are distinguished by the way of the antigen-antibody reaction recorded.

Classical enzyme-linked immunosorbent assay uses immobilized monoclonal antibodies against IMA to target the detection of a modified albumin N-terminus. Findings of measurement per the protocols of different ELISA manufacturers are represented in ng/mL and give comparable information about IMA content in normal serum [55–57]. It should be noted that no correlation has been found between the ACB assay and ELISA, accurately detecting the N-terminal modification of albumin in patients with the acute coronary syndrome (ACS) or nonischemic chest pain [51]. This is consistent with data showing that metal-binding sites A and B play a more critical role in cobalt binding than the N-terminus [36, 37].

A few types of biosensors for determining IMA have been developed in different laboratories. One of them is a liquid crystal biosensor (LCB) developed by He et al. [58], who immobilized anti-IMA on the surface of a glass slide and showed that the binding of the antibody with IMA induced orientation transformation of LCB and resulted in optical signal changes on the glass surface. According to their pilot results, the proposed sensor combined the high specificity of the antibodies with a sensitive optical signal amplification of LCB molecules, simplicity to operate, and low cost. The sensitivity of this sensor is reportedly less than 50 *μ*g/mL.

A novel type of sensor based on assembling anti-IMA onto an AuNP-modified gold chip was explored and used by Li et al. [59] to construct a Surface Plasmon Resonance (SPR) immune sensor for IMA evaluation. Compared with a direct binding SPR assay at a 100 ng/L limit of detection, gold nanoparticles (AuNPs) dramatically improved the sensitivity of IMA detection to 10 ng/L. Such sensors can provide a considerable increase in the sensitivity of analysis and enable testing *in vivo* and the identification of ІМА localization; however, they must be further developed and tested clinically before using in laboratory diagnostics.

Luo et al. proposed a new strategy for interference-free, simple, and rapid evaluation of IMA concentration, namely, quantum dot- (QD-) coupled X-ray fluorescence spectroscopy (Q-XRF) [60]. The proposed approach combines a high-specific sandwich immunoassay with the sensitivity of XRF spectroscopy. In a typical Q-XRF assay, serum total HSA is quantified using quantum dot-coupled sandwich immunoassay, and intact HSA (iHSA) is determined using XRF spectroscopy, by measuring the XRF intensity of Co^2+^ bonded to iHSA. IMA concentration is automatically determined by calculating the difference between total HSA and iHSA. The authors emphasize that the proposed Q-XRF assay, which integrates the classical microplate sandwich immunoassay, XRF spectrum assay, and QD labeling, can readout IMA concentrations in an extraordinarily rapid and accurate manner. A comparison between Q-XRF findings and results of the ACB test showed that Q-XRF had higher sensitivity than ACB, and its lowest detection limit was 0.05 U/mL, while ACB's was about 0.1 ABSU (our unpublished data). Per Luo and coauthors, the most significant improvement in the Q-XRF assay is its ability to accurately detect true IMA values, regardless of interferences from extremely high or low albumin concentrations or influence of bilirubin. Taking into account the peculiarities of the structural organization of albumin and the available evidence of allosteric inhibition of its metal-binding sites by FA, this conclusion should be experimentally verified [61].

## 4. ІМА in Physiological Hypoxia

Physical activity is accompanied by metabolic changes that cause ischemia in muscle tissue. Ischemia, induced by moderate physical activity, is usually short-term and is generally resolved without significant consequences. However, Falkensammer et al. found that exercise-induced calf muscle ischemia in healthy individuals is accompanied by an increase in IMA levels in serum, which return to baseline within 30 minutes. An investigation of myocardial markers under this condition did not show any significant association between changes of IMA and levels of lactate, cardiac troponin T (cTnT), and an N-terminal fragment of brain natriuretic peptide (NTproBNP) after ischemia [41].

Other findings have been reported in untrained individuals after induced forearm ischemia. A low IMA level under an increase of lactate was recorded at the beginning of an exercise stress test, but it was restored to baseline values in 1-5 minutes after the test [39]. Regular endurance exercises contribute to an increase in blood IMA, correlating with increased levels of standard cardiac markers, including lactate dehydrogenase (LDH) and creatine kinase MB (CK-MB), compared with people who lead a sedentary lifestyle [43]. According to Apple et al., the dynamic of changes in ІМА levels in long-distance runners is slightly different from that under conditions of minimal or moderate physical activity. Prolonged skeletal muscle ischemia in athletes after a marathon is accompanied by a decrease in IMA levels, which, in 63% of cases, goes back up to the baseline values in 24 to 48 hours after the marathon [38]. Other authors did not find significant IMA level changes in athletes after 12 h and 24 h long runs despite significantly elevated LDH, CK-МВ, сTnT, and NT-proBNP. Because all values return to normal after 48 hours, there are suggestions that endurance exercises cause acute but transient cardiac dysfunction, and the duration and intensity of the exercises play an essential role in adapting to temporary ischemia [62].

Oxygen deficiency is also observed in association with metabolic changes during normal pregnancy. Physiological hypoxia at the early stages of trophoblast development determines its invasive and proliferative properties [63]. An increased generation of reactive oxygen species also occurs during pregnancy, leading to the oxidative damage of the trophoblast and the initiation of fetal membrane formation [64]. In this context, the level of IMA in pregnant women is almost two times higher than in nonpregnant women and correlates with the level of thiobarbituric acid reactive substances (TBARS) [65].

## 5. ІМА as a Marker for Diseases

IMA has been proposed as an early biomarker for various diseases associated with ischemia and oxidative stress, including myocardial infarction and cerebrovascular accidents, diabetes mellitus and renal failure, and hypothyroidism and hyperthyroidism [21, 23, 42, 66]. Mounting experimental data suggest that this marker is ambiguous and that its values depend on the type and stage of the pathological process and methods used. The clinical significance of IMA in diseases and the impact of different factors on its level are discussed below.

### 5.1. Cardiovascular Diseases

Cardiovascular diseases, especially acute coronary syndromes (ACS), are the leading causes of morbidity and mortality in humans worldwide. According to the World Health Organization (WHO), an estimated 17.5 million people die from cardiovascular diseases annually, and about 42% of these deaths are due to ACS [67, 68]. The advances in the study of heart diseases have led to the discovery of a broad range of novel biomarkers associated with cardiovascular risks, including cTnI and cTnT, B-type natriuretic peptide (BNP) and its prohormone NT-proBNP, C-reactive protein (CRP), myeloperoxidase (MPO), lipoprotein-associated phospholipase A2, miRNA, matrix metalloproteinases, and cystatin C [69, 70]. Although these biomarkers have a prognostic value independent of the previous traditional risk factors, they have some limitations as early markers for ACS, including unstable angina, non-ST-segment elevation myocardial infarction (NSTEMI), and ST-segment elevation myocardial infarction (STEMI) [69, 71].

As mentioned above, IMA was proposed as an early diagnostic indicator of myocardial infarction in 2000 [29]. Subsequently, Christenson et al. showed in a multicenter study that IMA was a potential earlier ACS predictor than cTnI. They examined 226 patients who arrived at the emergency departments (ED) within 3 hours of the onset of signs and symptoms suggestive of ACS. All the patients had negative cTnI at presentation, and this marker for necrosis started to increase within 6-24 hours of hospitalization. Findings showed that sensitivity and specificity for the ACB test were 70% and 80%, respectively, with a negative predictive value of 96% [72]. Other studies have supported these results. Chawla et al. found IMA's sensitivity and specificity for detecting ACS to be 78.0% and 82.7%, respectively, compared to 58.0% and 60.0% for the CK-MB assay [73]. Lee et al. obtained other results, finding the sensitivity and specificity of IMA for identifying ACS to be 93% and 35.6%, respectively, and the negative and positive predictive values to be 91.8% and 39.6%, respectively. The combination of myoglobin, CK-MB, and TnT demonstrated 80.2% of sensitivity and 57% of specificity for ACS diagnosis. Sensitivity increased to 94.5%, and specificity fell to 45.1% when IMA was included in the cardiac marker panel [44].

Many authors find IMA a more sensitive indicator of ACS than TnI, myoglobin, and CK-MB. These latter markers are informative only within 2-6 hours of the onset of chest pain and acute cardiac events, whereas IMA rises within 30 min and continues to increase for the next 6-12 hours [11, 74, 75]. IMA measurements allow for ACS speculations in the absence of changes in the electrocardiogram and unchanged cardiac markers [76]. A meta-analysis of the combination of ECG, Tn, and IMA in more than 1800 patients with ED demonstrated a high negative triple test (NTT) diagnostic value for excluding ACS. NTT sensitivity was 97.1% for ACS at the early stage and 94.5% for longer-term outputs [77]. However, an increased IMA level does not differentiate between ischemic and nonischemic chest pain or ACS types because its levels have not been shown to differ between patients with MI and those without MI. On the contrary, the means of total CPK, CK-MB, and TBARS have been revealed to increase in patients with MI [78].

Comparing results of the ACB test in three categories of ACS (STEMI, NSTEMI, and unstable angina) revealed that IMA tended to increase in the more severe disease, but the difference between the three groups was not statistically significant (Table 2) [79, 80].

Evaluation of serum IMA is recommended not only for early detection of myocardial ischemia but also as a prognostic indicator of the disease severity. People with higher IMA showed longer hospitalization days and had more readmissions as compared to patients with high troponin. However, top high-level IMA did not predict negative cardiovascular events during the hospital stay, while the cTnT test predicted arrhythmia more often than the ACB test [81].

Recently, there have been works on IMA's prognostic significance in acute aortic dissection (AAD). This fatal aortic sickness has a high death rate, demanding prompt examination and treatment [82]. About 1 to 2% of patients with AAD die per hour for the first 24-48 hours [83], making it crucial to have a reliable disease indicator. Eroglu et al. reported increasing IMA in patients with aortic dissection compared to healthy individuals [84]. They explained these results using generalized tissue hypoxia caused by aortic dissection and hemodynamic violations. A retrospective analysis of electronic health records in 731 AAD patients revealed a link between the level of IMA and in-hospital mortality. The specificity and sensitivity of IMA determining for in-hospital mortality in case of IMA levels ≥ 79.35 U/mL were 80.6 and 84.8%, respectively. Therefore, IMA is potentially an independent prognosticator for in-hospital mortality among AAD patients [85].

IMA levels also change after coronary artery bypass grafting. During the first hour postsurgery, IMA drastically decreases and then gradually returns to the baseline level within the next 48 hours. However, the absence of a significant difference in IMA values does not allow prestratifying patients for the risk of perioperative myocardial infarction [86].

Elevated IMA levels have also been observed in patients with chronic ischemic heart failure (CIHF) having a left ventricular ejection fraction (LVEF) of less than 45% [87]. Researches have demonstrated that IMA increases 2.4 times in patients with acute decompensated HF, resulting in less than 35% LVEF. It has also been suggested that in-hospital acute HF therapy significantly reduces IMA levels. These findings are consistent with Ellidag et al.'s data showing elevation in IMA and indicators of oxidative stress in patients with CIHF against the background of decreasing albumin content. The authors did not find IMA levels to correlate with the severity of the disease although albumin levels reduced more in severe HF. Based on these results, the authors proposed IMA as a promising biomarker for acute ischemia and albumin-dependent oxidative stress even this marker does not reflect the stage of heart damage [88].

Other results were obtained in patients with dilated cardiomyopathy (DCM). In their study, Sbarouni et al. found no significant difference in IMA levels between age-matched healthy volunteers and patients with compensated chronic heart failure (CHF) due to DCM. IMA levels in stable DCM patients did not differ among patients with positive and negative cTnI, suggesting that myocardial necrosis in compensated patients occurs without preceding transient ischemia [89]. DCM, which results from chemotherapy with anthracycline antibiotics, was also accompanied by an increase in IMA, and serum IMA levels correlated positively with the cumulative dose of an anthracycline antibiotic [90]. It was noted that epirubicin treatment leads to a more significant increase in IMA levels over time compared with doxorubicin treatment (195.6 ± 81.3 and 140.1 ± 14.8 U/mL, respectively) [91].

Thus, the analysis of literature on the diagnostic significance of IMA in cardiovascular pathology indicates that IMA alone has a limited value to the differential diagnosis of heart diseases. However, measuring IMA alongside other standard markers for myocardial damage and electrocardiography could provide an effective tool for early diagnosis and timely cardiac care and help predict long-term heart failure.

### 5.2. Neurological Disorders

Oxidative stress in the brain causes neurochemical and neuroanatomical changes and may play a role in developing many psychiatric disorders and neurodegenerative diseases, such as schizophrenia, Alzheimer's disease, Parkinson's disease, and Huntington's disease [92, 93]. High brain sensitivity and vulnerability to ischemia are associated with increased oxygen and glucose utilization and are also linked to large amounts of polyunsaturated fatty acids and metal ions in nervous tissues [94, 95]. Oxidative stress increases the formation of oxidative modified proteins (OMP). [16, 19]. Prolonged ischemia causes inflammation reactions, decreases ATP production by mitochondria, disrupts energy-dependent functions of the brain cells, including ion pumps, and damages the blood-brain barrier (BBB). One can expect that the larger the lesion volume, the more intense the inflammation reaction and loss of brain barrier integrity, leading potentially to an increased release into the systemic circulation not only of classical neuronal biomarkers, like S100, NCAM, or GFAP, but also OMP, including IMA [96, 97]. Therefore, changes in the level of IMA in the blood may reflect the degree of BBB damage in neurological disorders (Table 3).

Many clinical investigations have shown that IMA levels increase in the blood of patients with a stroke. Abboud et al. revealed that IMA levels increased in patients with brain infarction over 24 hours [104], and Gunduz et al. showed that IMA concentration in an ischemic stroke was 1.6 times higher than in control individuals [105]. According to current data, IMA content in the blood of patients with an ischemic stroke increases during the acute phase, followed by a gradual decrease within a week [54]. IMA's determination in the blood also allows ischemia to be differentiated from a hemorrhagic insult, as its level is significantly higher in patients with an ischemic stroke [106]. Okda et al. determined IMA's value for an ischemic stroke as 105.01 ± 10.81 U/mL vs. 99.24 ± 12.89 U/mL for intracerebral hemorrhage and 97.74 ± 13.36 U/mL for subarachnoid hemorrhage. They found a positive correlation between serum IMA levels and brain lesion volume, as calculated using the National Institute of Health Stroke Scale (NIHSS) and computed tomography data [98]. Many studies have proven that the IMAR is a more sensitive marker for a stroke than IMA [107–109]. According to a ROC analysis of IMAR to diagnose a stroke in one study, the area under the curve (AUC) was 0.990 (cut-off value 91.4; 95% CI: 0.970-1.000; sensitivity: 96.4%; specificity 95.8%), and the AUC for IMA was 0.928 (cut-off value 98 U/mL; 95% CI 0.857-0.999; sensitivity 89.3%; specificity 88.5%) [107].

The monitoring of IMAR during an in-hospital stay is predictive not only for a stroke but also for hemorrhagic complications. Randomized controlled experimental studies in Turkish health institutions showed that IMA and IMAR levels, general oxidative status, and oxidative stress index are increased in a hemorrhagic shock, and these changes depend on the duration of the shock. The most significant changes were identified in IMA and IMAR such that these indicators may be employed in the early diagnosis of hemorrhagic shock and as predictors of disease severity [109].

Because albumin is synthesized primarily in the liver, questions about IMA's origin in the serum of patients with ischemic brain injury are rife. Where exactly is IMA formed, and what causes an increase in modified albumin in peripheral circulation? Is the latter a result of BBB loss, or does the liver produce IMA in response to oxidative stress in the brain? Data on this area are limited due to the difficulty of obtaining material for analysis. The albumin content in nervous tissues is relatively low: its concentration in the cerebrospinal fluid (CSF) is about 0.2 g/L. However, the ratio of albumin to total CSF proteins here is 1.4 times higher than in plasma [3]. Initially, it was believed that albumin enters the brain from the blood and extracellular matrix by simple diffusion [110]. However, recent research has demonstrated that albumin can also be produced in the brain by microglial cells under certain circumstances, and the expression of albumin in microglial cells increases upon activation by lipopolysaccharide or amyloid protein A*β*_1-42_ [6]. *De novo* synthesis of hepatocyte nuclear factor-1 alpha (HNF-1*α*) has also been established in the rat brain, and it plays a role in the upregulation of albumin expression in focal brain ischemia [111].

On the other hand, activated microglia, astrocytes, and endothelial cells can absorb albumin and promote its elimination, thus protecting neurons from accumulating this protein [111, 112]. Given that albumin uptake by neurons is neurotoxic, rapid albumin clearance by microglia could prevent neuronal cell death. Therefore, the origin of IMA in the blood of patients with a stroke is under question. Comparing IMAR in the blood and CSF of patients with Alzheimer's disease (AD) showed a more significant increase in IMAR in the CSF [113]. Based on these data and the fact that only native albumin can cross the BBB, researchers have concluded that IMA in the CSF of patients with AD originates from the central nervous system. Increased IMA in the blood after brain infarction could result from oxidative stress during an acute stroke, leading to the production of OMP in the brain and their passage in circulation through the broken BBB [94–96].

An increase in IMA in the blood of patients with neurodegenerative diseases [113], psychiatric disorders [92, 93], and traumatic brain injuries [114] may be explained by the loss of integrity of the BBB associated with an increase in intracranial pressure (ICP). Various underlying mechanisms, including caspase and metalloproteinase activation, mitochondrial dysfunction, excessive glial activation, inflammatory reactions, and compromised microcirculation, are associated with increased ICP [115–117]. In line with that, Kara et al. have shown that increased intracerebral pressure in rats due to injury, hematoma, or cerebral edema leads to increased IMA levels in the blood [118]. Moreover, IMA levels correlate with the amount of TBARS in the blood, and both can serve as additional markers for increased intracerebral pressure and can be valuable in predicting brain death. These results are consistent with clinical follow-up data of patients with traumatic brain injury of variable severity, which showed that the level IMA could predict mortality with sensitivity and specificity from 81.8 to 100% [114].

IMA's diagnostic value in psychiatric disorders, such as AD, schizophrenia, and bipolar disorders, is controversial [101, 102]. An increase in IMA, IMAR, and some markers for oxidative stress, such as advanced oxidation protein products (AOPP), ceruloplasmin, and prooxidant-antioxidant balance (PAB), has been shown in AD, the most common form of dementia in the elderly population [99, 119]. Multivariate analysis revealed that a serum IMA level of ≥476.4 ng/mL and an IMA/albumin ratio of ≥9 are separately associated with the development of mild cognitive impairment in AD patients [120]. Increased IMA levels have also been found in patients with major depressive disorder. One study reported a positive correlation between the severity of depression and IMA levels [100], but another study did not detect a significant difference between IMA levels in the serum of patients with bipolar disorder during remission and healthy controls [102]. In contrast, Tunç et al. detected increased IMA levels in bipolar disorder patients during remission, although they found no IMA changes in unipolar depression patients [101].

Neonatal hypoxic-ischemic encephalopathy (HIE) is a common disease caused by perinatal asphyxia, a major cause of neonatal death, neurological behavior, and long-term disability. Currently, the diagnosis and prognosis of neonatal HIE are based on clinical manifestations of neurological disorders, electrophysiological examination, and the use of brain-related biomarkers, including NSE, S-100*β*, GFAP, tau protein, miRNA, LDH, and CK-BB. Their determination take time, and late diagnosis of brain injuries occur in newborns so that many infants miss the ideal treatment time and are left with varying degrees of neurological sequelae [121]. The determination of IMA in umbilical cord blood opens up new perspectives in diagnosing and predicting possible neurological complications in infants. Recent studies have shown that umbilical cord blood IMA levels are higher in neonates with neonatal encephalopathy than in healthy infants (250.83 ± 36.07 pmol/mL vs. 120.24 ± 38.9 pmol/mL). A comparison of IMA content in groups of infants with varying degrees of cerebral hypoxia revealed significant differences between mild, moderate, and severe degrees of hypoxia groups at 207.3 ± 26.65, 259.28 ± 11.68, and 294.99 ± 4.41 pmol/mL, respectively. The diagnostic value of IMA depends on the threshold value selected for calculation. For example, at a threshold of 197.6 pmol/mL, sensitivity was 84.5%, specificity was 86%, the positive prognostic value was 82.8%, and the negative prognostic value was 88.3% [103]. A recent study showed that IMA might be a novel marker for predicting neonatal neurologic injury in small-for-gestational-age infants in addition to neuron-specific enolase [122]. It was, therefore, suggested that IMA levels could be used as markers for the diagnosis of ischemic encephalopathy in the early postnatal period and as a predictor of delayed posttraumatic neurological complications in children.

### 5.3. Diabetes Mellitus and Its Complications

Chronic hyperglycemia in patients with diabetes mellitus (DM) is accompanied by the progression of oxidative stress- and hypoxia-induced ischemia. Prolonged endothelial cell exposure to hyperglycemia stimulates protein glycation, the formation of highly reactogenic by-products, and the activation of lipid peroxidation. In addition, a decrease in the bioavailability of nitric oxide (NO), an imbalance between vascular endothelial growth factor (VEGF) and NO, increased synthesis of proinflammatory cytokines, abnormal angiogenesis, and impaired endothelial regeneration occur in DM. These processes and metabolic changes result in the development of acute (diabetic ketoacidosis) and chronic complications of DM (nephrosis, peripheral vascular insufficiency, neuropathy, etc.) [123, 124].

Analyses of clinical and experimental investigations have shown that types one and two DM (DM1 and DM2) are associated with an increase in IMA, the level of which depends on glucose concentration, presence of complications, and comorbidities [125–129]. On the other hand, the formation of IMA in the early stages of DM plays a significant role in the pathogenesis of diabetic complications. For a long time, the most sensitive marker for diabetic ketoacidosis in patients with DM1 was declared CRP [130]. Last studies suggest the use of IMA as an independent marker for impaired glucose metabolism. According to Ma et al., this indicator correlates with CRP and glucose levels in uncomplicated DM1 and diabetes with ketoacidosis; however, IMA levels are much higher in patients with ketoacidosis. It should also be noted that IMA is quite sensitive to insulin therapy which quickly normalizes IMA levels in the blood of diabetic patients [125].

Hyperglycemia-induced ischemia, inflammation, and oxidative stress might increase IMA levels not only in the serum but also in the kidney, resulting in podocyte malfunction. Their excess accumulation along the extracellular matrix in the glomerulus and tubulointerstitium leads to vascular endothelial damage and the development of diabetic nephropathy (DN) [131, 132]. Dash et al. showed that IMA, as a marker, might help determine underlying subclinical diseases or vascular dysfunction in a diabetic kidney [133]. A significant difference in plasmas' IMA was found among patients with early DN, diabetes without nephropathy, and healthy controls, with the highest levels in early DN. The upsurge of IMA level in the plasma of patients with DN correlated with changes in AOPP, HbA1c, urine albumin/creatinine ratio (UACR), and serum creatinine [55]. According to the results of prospective analysis, an increase in the level of glycated hemoglobin (HbA1c) is always accompanied by an increase in IMA levels [123, 134]. A similar pattern of changes has been observed in diabetes complicated by retinopathy, with IMA correlating with fasting glucose content and HbA1c levels against a background of low glutathione content [135, 136].

There has been no identified relationship between urine IMA levels and microalbuminuria associated with diabetic nephropathy and other DM complications, indicating that IMA evaluation has a higher diagnostic significance in the blood [42]. Further studies on the urinary excretion mechanisms of IMA must be conducted to use urinary IMA levels as a diagnostic marker for DM complications.

Data on the relationship between IMA and lipid profiles in DM complications are controversial. Previous research found a positive correlation between the levels of IMA, total cholesterol, and low-density lipoprotein (LDL) in patients with diabetic nephropathy [134]. Subsequently, it was revealed that IMA levels rise in patients with nephropathy and retinopathy against the background of low levels of high-density lipoprotein. In opposite, patients with increased LDL had a low IMA content in their plasma [123, 136]. An increase in IMA in DM2 could indicate subclinical vascular diseases: the baseline values of IMA in patients with peripheral vascular lesions are much higher than in patients without cardiovascular disorders [137]. Thus, IMA is not only an additional criterion for glycemic control and the early detection of diabetic complications but also can be a prognostic marker for peripheral vascular lesions.

### 5.4. Obstetrics and Gynecology

Pregnancy develops in a relatively hypoxic intrauterine environment and is accompanied by the generation of ROS, causing oxidative damage to the trophoblast and initiating the formation of the fetoplacental complex [63, 64, 138]. Under these conditions, an increase in the IMA levels in amniotic fluid and maternal and umbilical cord blood should be expected [139, 140]. Experimental and clinical studies have confirmed that maternal blood IMA levels increased at the early stages of normal pregnancy and in the third trimester of normal pregnancy. During normal pregnancy, maternal blood serum IMA levels increase by almost 2 times in the first trimester of pregnancy and continuously remain high throughout the entire gestational period [65]. In the event of complications at any stage of gestation, during childbirth, or postpartum, maternal serum IMA levels significantly (Table 4).

Manifestations of nausea and severe vomiting (*Hyperemesis gravidarum*) in pregnant women are accompanied by a 1.2 times increase in IMA concentration compared to healthy pregnant women [141]. Pregnancy complications with fetal growth retardation due to placental insufficiency at the end of the second trimester and after delivery are accompanied by a 1.5-fold increase in the maternal serum IMA levels compared to normal pregnancy [150].

Increased IMA concentrations are also observed in spontaneous abortions in women during early pregnancy. To stratify healthy pregnant women and women with pregnancy loss in the first trimester, the prognostic value of IMA was determined using a threshold value of >163 ng/mL, with 75% sensitivity and 55% specificity [142]. However, the simultaneous determination of IMA level, free *β*-chorionic human gonadotropin, and progesterone with the same sensitivity yielded the 99% specificity in the prediction of spontaneous abortion in women with a history of recurrent miscarriages [151]. Recently, the evaluation of AAIMA and its use to calculate the index of oxidation (IOS) was proposed as a marker of ectopic pregnancy. According to Bozkaya et al., a cut-off value of 0.545 for IOS has 81.6% sensitivity and 59.5% specificity for the prognosis of ectopic pregnancy [144]. This indicator can be especially important during an artificial insemination, as the frequency of ectopic pregnancy and spontaneous abortions in such patients remain high [152].

Abnormal intrauterine hypoxia in the early stages of pregnancy can cause oxidative damage to the trophoblast during reperfusion, contributing to the classic symptom complex (proteinuria, hypertension) of preeclampsia (PE) after the 20th week of pregnancy. PE is a leading cause of maternal and perinatal morbidity and mortality; therefore, the search for prognostic markers of this pregnancy complication is highly relevant. Increased oxidative stress and decreased antioxidant protection in PE are associated with a sharp increase in the level of IMA in mothers' blood. Increased IMA during the first trimester has been shown in pregnant with premature preeclampsia and then proposed as an early indicator of this complication [146]. This indicator returns to normal within 48 hours of birth, with symptoms of preeclampsia disappearing [147]. A meta-analysis of publications available in NCBI PubMed and other databases conducted by Reddy et al. [153] showed that IMA is a potentially useful biomarker for PE with reasonable accuracy (AUC = 0.860). The authors concluded that the evaluation of maternal serum IMA and fetal cord-blood IMA concentrations is useful as a simple, novel, and inexpensive marker of oxidative stress status in PE patients [154].

The relationship between IMA and gynecological disorders has been studied extensively for the last 10 years. Detailed analysis of literature data on IMA changes in the blood and follicular fluid due to gynecological pathologies, such as dysmenorrhea, endometriosis, polycystic ovary syndrome (PCOS), uterine artery embolization, menopause, and infertility, is presented in the review of Kıncı et al. [155]. Based on the analysis, the authors concluded as follows: (i) serum IMA may be useful in evaluating dysmenorrhea severity; (ii) follicular IMA provides information about the state of oocytes and embryo quality in vitro fertilization; (iii) the diagnostic significance of IMA in polycystic ovary syndrome is contradictory and require further research. Reddy et al. conducted a meta-analysis of nine studies and showed that the serum IMA levels were increased significantly in PCOS patients compared to non-PCOS controls [156]. The authors concluded that IMA is potentially a reliable and novel marker reflecting increased oxidative stress in PCOS. Conclusively, the data, discussed in this section, suggest that this promising marker could be used for early diagnosis and follow-up of gynecological diseases associated with oxidative stress.

### 5.5. Cancer

Oxidative stress persistently accompanies many cancer types due to increased production of ROS and reactive nitrogen species (RNS) or to reduced effectiveness of the antioxidant system. The effects of increased ROS and RNS vary according to their radical forms, concentrations, and where they occur, but they affect cancer cells by triggering DNA damage, stimulating genetic mutations, and inhibiting apoptosis, influencing proliferation, invasion, and metastasis [157]. Therefore, the antioxidant/oxidative parameters of tumors are prognostically crucial in many types of cancer. These parameters can be assessed via the detection of well-known oxidative markers of proteins, such as protein carbonyls, advanced oxidation protein products, and IMA [158]. Many investigations have demonstrated that IMA levels significantly increased in different proliferative diseases (Table 5).

According to the ROC analysis, IMA has sensitivity above 80% as an auxiliary biomarker in the diagnosis of such diseases as breast and colon cancer [158]. It can be effective in demonstrating hypoxia in patients with acute leukemia [166] and used as an additional indicator for the prognosis of the myelodysplastic syndrome [167]. Most of the researchers consider that the evaluation of IMA levels to be a reliable biomarker of oxidative stress reflecting tumor ischemia; however, only detailed studies in which the steps of carcinogenesis are examined one after the other in terms of oxidative stress and antioxidant activity can confirm this possibility.

## 6. Conclusion

In summary, this review of the currently available literature suggests that IMA is a nonspecific marker for many diseases, which are tightly associated with ischemia and oxidative stress. Despite this marker's low specificity, the evaluation of its content may provide valuable information regarding the duration of diseases and possible complications, and it can be used in the differential diagnosis of certain pathological conditions. IMA's advantage as a biomarker over other markers is its ability to detect ischemic conditions at earlier stages. The simplicity and availability of the techniques for its determination provide an opportunity to stratify patients and determine risk groups for adverse events after a stroke, heart attack, traumatic brain injuries, and spinal injuries and assess the state of patients with neurological disorders, diabetes, pregnancy complications, and with gynecological and other ischemic-associated pathologies.

## Figures and Tables

**Table 1:** The comparison of methods for IMA measuring.

Method	Average content in control	Advantages	Limitations	Ref.
Colorimetric methods				
ACB	0.39-0.43 ABSU 79 ± 6.3 U/mL	Simple, low cost, automation possibility	Dependent on pH, temperature, level of HSA and free FA, the proportion of the intact HSA N-terminus, the state of cys34 oxidation in HSA, lack of standardization	[23, 29, 51, 52, 54]
CAB	0.53 ± 0.04 ABSU	Affordable and simple, small sample volume, the possibility to analyze the structural differences of HSA, independent of HSA concentration	Dependent on HSA and free FA concentrations, on the proportion of the intact N-terminus of HSA, lack of standardization	[53]
NAB	0.415 ± 0.084 ABSU	More sensitivity than ACB	Not widespread, not enough information	[48, 49]
ACuB	There is no data	More accurate than ACB, highly reliable, and highly sensitive	Poorly developed not enough information	[50]

Immunochemical methods				
ELISA	45.7 ± 23.9 ng/mL 62.21 ± 21.47 ng/mL 43.4 ng/mL (1.1–320.3)	High sensitivity and specificity for NTS	Cost analysis, high antibody affinity	[55] [56] [57]
LCB	50 *μ*g/mL	Simple, does not require measuring technology	The high cost of the biosensor and the lack of its production, low sensitivity and accuracy	[58]
SPRI	10-100 ng/L	High sensitivity and specificity	Availability of appropriate equipment, no clinical trials	[59]
Q-XRF	0.05 U/mL		Availability of appropriate equipment, no clinical trials	[60, 61]

Note: ACB: Albumin Cobalt Binding test; ACuB: Albumin Copper Binding assay; CAB: Cobalt-Albumin Binding test; ELISA: enzyme-linked immunosorbent assay; FA: fatty acids; LCB: liquid crystal biosensor; NAB: nickel-albumin binding assay; SPRI: surface plasmon resonance immunosensor; Q-XRF: X-ray fluorescence spectroscopy; ABSU: absorbance units; U/mL: units in liter (one unit was defined as of free Co^2+^ in the reaction mixture per mL of serum sample.

**Table 2:** Changes of IMA content in serum of patients with cardiovascular diseases.

Pathology		Age, years	No of examined	IMA value		Combination with other markers and sensitivity	Ref.
				Control	Patients		
Acute coronary syndrome	NSTEMI STEMI UA	62.32 ± 16.63	*n* = 50	0.410 ± 0.081 ABSU	0.925 ± 0.094 ABSU 0.843 ± 0.146 ABSU 0.783 ± 0.221 ABSU	cTnI, CK-MB, ECG 92-94%	[79]
	NSTEMI STEMI UA	—	*n* = 135	54.70 ± 17.29 U/mL	87.31 ± 5.95 U/mL 92.10 ± 10.60 U/mL 88.90 ± 6.16 U/mL	cTnI, CK-MB 88%	[80]

Acute aortic dissection		53 ± 7	*n* = 98	0.62 ± 0.18 ABSU	0.70 ± 0.13 ABSU	cTnT, CK-MB 84.7%^∗^	[84]
		52.99 ± 12.17	*n* = 731	—	74.66 ± 20.84 U/mL	IMA–independent forecaster for in-hospital mortality	[85]

Chronic heart failure		68 ± 7	*n* = 59	0.379 ± 0.08 ABSU	0.894 ± 0.23 ABSU	cTnI, NT-proBNP 92.9%^∗^	[87]
		70 ± 11	*n* = 55	0.470 ± 0.1 ABSU	0.669 ± 0.2 ABSU	Total antioxidant status, total oxidant status, oxidative stress index–not correlation	[88]

Dilated cardiomyopathy		46 ± 14	*n* = 42	93.9 ± 9.9 (76-122) kU/L	89.9 ± 13.1 (71-117) kU/L	cTnI, CK-MB, CPK, NT-proBNP, total protein, albumin Not significance	[89]
		56 (range 35-68)	*n* = 152	Prechemotherapy 59.2 ± 10.9 U/mL	After the sixth cycle of chemotherapy 140.1 ± 14.8 U/mL	cTnT, CK-MB 92%	[90]

Note: IMA was measured in serum by ACB assay. ^∗^ = sensitivity for IMA alone; ABSU: absorbance units; U: units; CPK: creatine phosphokinase; CK-MB: creatine kinase MB; cTn: cardiac troponin; ECG: electrocardiogram; NSTEMI: non-ST-segment elevation myocardial infarction; STEMI: ST-segment elevation myocardial infarction; NT-proBNP: N-terminal prohormone of brain natriuretic peptide; UA: unstable angina.

**Table 3:** Changes of IMA in neurological disorders.

Pathology	Age, years	No. enrolled, method	IMA value		Reference
			Control	Patients	
Acute ischemic stroke	55-56	*n* = 50, ACB	70.71 ± 8.42 U/mL 79 ± 6.3 IU/L 44.47 ± 5.28 U/mL	97.56 ± 13.74 U/mL 108 ± 8.9 IU/L 96.83 ± 12.01 U/mL	[95] [54] [98]

Alzheimer's disease	79.68 ± 7.58	*n* = 38, ELISA	409.59 ± 66.35 ng/mL	609.17 ± 327.61 ng/mL	[99]

Depression	39.4	*n* = 59, ACB	0.73-0.90 ABSU	0.66–0.92 ABSU	[100]

Schizophrenia	33.61 ± 10.02	*n* = 28, ACB	0.44 ± 0.09 ABSU	0.53 ± 0.15 ABSU	[101]

Bipolar disorder (BD)	33.34 ± 1.13	*n* = 32, ACB	0.44 ± 0.09 ABSU	0.54 ± 0.16 ABSU	[101]

BD in remission	38.2 ± 7.5	*n* = 35	0.546 ± 0.13 ABSU	0.532 ± 0.14 ABSU	[102]

Neonatal hypoxic-ischemic encephalopathy (HIE)	Neonates^∗^	*n* = 60, ELISA *n* = 18 *n* = 30 *n* = 12	120.24 ± 38.9 pmol/mL	250.83 ± 36.07 pmol/mL 207.3 ± 26.65 pmol/mL (mild), 259.28 ± 11.68 pmol/mL (moderate), 294.99 ± 4.41 pmol/mL (severe degree)	[103]

^∗^IMA levels in cord blood.

**Table 4:** IMA changes in the serum of pregnant women with different complications.

Pathology	No. enrolled, method	IMA value		Reference
		Normal pregnant women	Study group	
*Hyperemesis gravidarum*	*n* = 45, ELISA	6.9 ± 0.3 ng/mL	8.2 ± 0.2 ng/mL	[141]

First trimester abortions	*n* = 60 *n* = 45, ELISA	Median 173.2 ng/mL Range 94.6–451.2 Median 43.4 ng/mL Range 1.1–320.3	Median 206.5 ng/mL Range 28.7–775.3 Median 63.7 ng/mL Range 20.1–285.2	[142] [57]

Recurrent first trimester abortions	*n* = 43, ACB	0.88 ± 0.10 ABSU	1.11 ± 0.08 ABSU	[143]

Ectopic pregnancy	*n* = 38, ACB	0.484 ± 0.089 ABSU	0.577 ± 0.117 ABSU	[144]

Hypertensive pregnancy disorders	*n* = 40, ACB	0.374 ± 0.114 ABSU	0.465 ± 0.154 ABSU	[145]

Preterm preeclampsia	*n* = 19, ACB	Median 115.01 kU/L Range 102.29–124.81	Median 126.5 kU/L Range 114.33–134.36	[146]

Preeclampsia	*n* = 57, ACB *n* = 47, *n* = 45	0.77 ± 0.24 ABSU 0.76 ± 0.07 ABSU 71.61 ± 09.58 U/mL	1.24 ± 0.30 ABSU 0.80 ± 0.07 ABSU 106.92 ± 15.20 U/mL	[147] [148] [149]

**Table 5:** Changes of IMA in proliferative diseases.

Pathology	No. enrolled, method	IMA value		Reference
		Control	Study group	
Breast cancer	*n* = 127, ACB	Median 0.62 ABSU Range 0.19–1.31	Median 0.66 ABSU Range 0.31–3.30	[159]
	*n* = 45, ELISA	452.05 ± 61.05 ng/mL	527.85 ± 131.02 ng/mL	[158]

Endometrial cancer	*n* = 43, ACB	Median 0.490 ABSU Range 0.407–0.589	Median 0.489 ABSU Range 0.401–0.611	[160]

Prostate cancer	*n* = 64, ACB	0.443 ± 0.49 ABSU	0.843 ± 0.76 ABSU	[161]

Bladder cancer	*n* = 30, ACB	0.474 ± 0.04 ABSU	0.588 ± 0.07 ABSU	[162]

Gastric cancer	*n* = 52, ACB	0.271 ± 0.066 ABSU	0.405 ± 0.111 ABSU	[163]

Colorectal carcinoma	*n* = 40, ACB	0.469 ± 0.04 ABSU	0.569 ± 0.06 ABSU	[164]
	*n* = 45, ELISA	452.05 ± 61.05 ng/mL	559.21 ± 140.03 ng/mL	[158]

Multiple myeloma	*n* = 40, ACB	0.369 ± 0.03 ABSU	0.555 ± 0.24 ABSU	[165]

Acute myeloid leukemia	*n* = 38, ACB	0.50 ± 0.09 ABSU	0.69 ± 0.14 ABSU	[166]
